# Improving Detection and Response to Respiratory Events — Kenya, April 2016–April 2020

**DOI:** 10.15585/mmwr.mm6918a2

**Published:** 2020-05-08

**Authors:** Osatohamwen I. Idubor, Miwako Kobayashi, Linus Ndegwa, Mary Okeyo, Tura Galgalo, Rosalia Kalani, Susan Githii, Elizabeth Hunsperger, Arunmozhi Balajee, Jennifer R. Verani, Maria da Gloria Carvalho, Jonas Winchell, Chris A. Van Beneden, Marc-Alain Widdowson, Lyndah Makayotto, Sandra S. Chaves

**Affiliations:** ^1^Epidemic Intelligence Service, CDC; ^2^Division of Bacterial Diseases, National Center for Immunization and Respiratory Diseases, CDC; ^3^Division of Global Health Protection, Center for Global Health, CDC; ^4^National Influenza Center Laboratory, National Public Health Laboratory, Ministry of Health, Kenya; ^5^Disease Surveillance and Response Unit, Ministry of Health, Kenya; ^6^National Public Health Laboratory, Ministry of Health, Kenya; ^7^Division of Viral Diseases, National Center for Immunization and Respiratory Diseases, CDC; ^8^Institute of Tropical Medicine, Antwerp, Belgium; ^9^Influenza Division, National Center for Immunization and Respiratory Diseases, CDC.

Respiratory pathogens, such as novel influenza A viruses, Middle East respiratory syndrome coronavirus (MERS-CoV), and now, severe acute respiratory syndrome coronavirus 2 (SARS-CoV-2), are of particular concern because of their high transmissibility and history of global spread (*1*). Clusters of severe respiratory disease are challenging to investigate, especially in resource-limited settings, and disease etiology often is not well understood. In 2014, endorsed by the Group of Seven (G7),* the Global Health Security Agenda (GHSA) was established to help build country capacity to prevent, detect, and respond to infectious disease threats.^†^ GHSA is a multinational, multisectoral collaboration to support countries towards full implementation of the World Health Organization’s International Health Regulations (IHR).^§^ Initially, 11 technical areas for collaborator participation were identified to meet GHSA goals. CDC developed the Detection and Response to Respiratory Events (DaRRE) strategy in 2014 to enhance country capacity to identify and control respiratory disease outbreaks. DaRRE initiatives support the four of 11 GHSA technical areas that CDC focuses on: surveillance, laboratory capacity, emergency operations, and workforce development.^¶^ In 2016, Kenya was selected to pilot DaRRE because of its existing respiratory disease surveillance and laboratory platforms and well-developed Field Epidemiology and Laboratory Training Program (FELTP) (*2*). During 2016–2020, Kenya’s DaRRE partners (CDC, the Kenya Ministry of Health [MoH], and Kenya’s county public health officials) conceptualized, planned, and implemented key components of DaRRE. Activities were selected based on existing capacity and determined by the Kenya MoH and included 1) expansion of severe acute respiratory illness (SARI) surveillance sites; 2) piloting of community event-based surveillance; 3) expansion of laboratory diagnostic capacity; 4) training of public health practitioners in detection, investigation, and response to respiratory threats; and 5) improvement of response capacity by the national emergency operations center (EOC). Progress on DaRRE activity implementation was assessed throughout the process. This pilot in Kenya demonstrated that DaRRE can support IHR requirements and can capitalize on a country’s existing resources by tailoring tools to improve public health preparedness based on countries’ needs.

## Improving Respiratory Disease Surveillance

**Expanding SARI surveillance.** SARI is defined as an acute respiratory illness requiring hospitalization, characterized by a subjective history of fever or measured temperature of 100.4°F (≥38°C) and cough, with onset within the past 10 days (*3*). Kenya currently has eight SARI surveillance sites. From these sites, nasal and throat swabs collected during Monday–Wednesday from patients who meet the surveillance case definition are sent to the National Influenza Center in Nairobi for influenza testing (Figure). In 2006, six of the eight surveillance sites were established in public health referral hospitals to monitor influenza disease trends (*4*). DaRRE partners expanded this surveillance network to include hospitals serving patients at increased risk for emerging respiratory diseases. In June 2017, Marsabit county was added to Kenya’s SARI network because of the county’s experience with a high prevalence of MERS-CoV seropositivity among camels (a natural reservoir host for MERS-CoV), which has been linked to human infections (*5*). In addition, the surveillance capacity at the refugee camp in Kakuma was strengthened by adding a trained surveillance officer and standardization of the case definition to be consistent with that used at other SARI sites. Because of the extensive air travel between Nairobi, Kenya, and the Middle East (where MERS-CoV has been reported) and China (where avian influenza A/H7N9 has been reported), SARI surveillance will be established in two large private hospitals in Nairobi during the current year to improve ascertainment of cases among international travelers, particularly persons who do not often seek care at public hospitals. Because of the ongoing coronavirus disease 2019 (COVID-19) pandemic, the DaRRE surveillance platform is being used for COVID-19 case detection.

**FIGURE:**
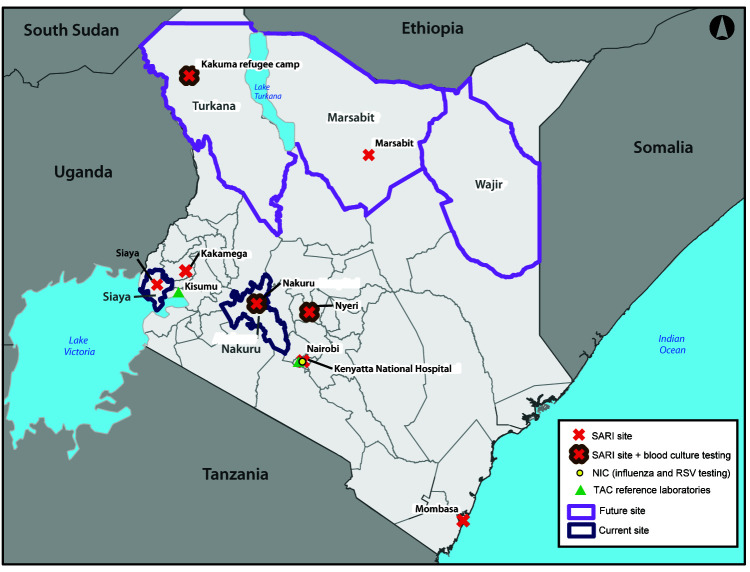
Current and proposed sites implementing severe acute respiratory illness (SARI)*^,†^ and event-based surveillance^§^ as part of the Detection and Response to Respiratory Events (DaRRE) strategy — Kenya, April 2020 **Abbreviations:** NIC = National Influenza Center; RSV = respiratory syncytial virus; TAC = TaqMan array card. * The eight hospital-based, sentinel SARI surveillance sites include Kakamega County Referral Hospital, Marsabit County Referral Hospital, Mombasa County Referral Hospital, Nakuru County Referral Hospital, Nyeri County Referral Hospital, Siaya County Referral Hospital, the Kenyatta National Hospital in Nairobi, and the refugee camp in Kakuma. ^†^ TAC diagnostic capacities are housed at KEMRI National Laboratory locations in Nairobi and Kisumu. ^§^ Five Kenyan counties selected for the event-based surveillance pilot program are Siaya and Nakuru (current sites) and Marsabit, Turkana, and Wajir (future sites).

**Piloting community event-based surveillance.** Event-based surveillance, defined as “the organized collection, monitoring, assessment, and interpretation of mainly unstructured ad hoc information regarding health events or risks, which may represent an acute risk to human health” (*6*), complements routine public health surveillance by including real-time reporting from multiple informal sources in the community (e.g., teachers, village health workers, community leaders, police, or media) (*7*). Event-based surveillance allows for detection of health events among populations often not included in routine surveillance, including certain groups of persons (e.g., refugees) and animals (e.g., birds). A mobile phone–based electronic reporting system to enable rapid reporting of events from the community level to the county and national levels has been developed and will complement the national EOC hotline system. Both systems were integrated into the EOC dashboard at the county and national levels for real-time reporting and response coordination. In March 2019, the first stakeholder meeting on community event-based surveillance in Kenya was held, engaging Kenya’s MoH and Ministry of Agriculture, Livestock and Irrigation, CDC, the U.S. Agency for International Development, Kenya Red Cross, the International Federation of Red Cross and Red Crescent Societies, Africa Centres for Disease Control and Prevention, and other partners to outline a unified strategy for community event-based surveillance in Kenya. In September 2019, a total of 397 community volunteers were trained in two pilot counties (Nakuru and Siaya). During the current year, three additional counties will be added as pilot sites, and DaRRE partners plan to train community volunteers on signals that focus on COVID-19 case detection.

## Enhancing Multilevel Diagnostic Capacity

DaRRE partners improved diagnostic capacity for identifying respiratory pathogens at both the national and county levels (Table 1). Because respiratory syncytial virus was known to be a leading cause of pneumonia resulting in hospitalization of children aged <5 years in low- and middle-income countries (*8*), molecular testing of SARI specimens for the virus at the National Influenza Center was initiated to provide further context to viral circulation within the country. A multipathogen diagnostic test using the TaqMan array card (TAC), available at the Kenya Medical Research Institute laboratories in Nairobi and Kisumu, was customized for respiratory disease outbreaks in Kenya and includes testing for MERS-CoV. TAC is a flexible platform that is currently being configured to detect SARS-CoV-2, among other high-impact targets, and is expected to be available in the next several weeks. To strengthen pathogen detection through blood culture, automated blood culture diagnostic systems and blood culture supplies were provided to the referral hospital laboratories in three counties and laboratory technicians were trained at these locations.

**TABLE 1:** Diagnostic capacity strengthening through implementation of the Detection and Response to Respiratory Events strategy — Kenya, April 2016–April 2020

Administrative level	Type of test	Pathogens
**National**		
National Influenza Center, Nairobi	PCR	Influenza (detection and subtyping)
		Respiratory syncytial virus
KEMRI Centre for Global Health Research Laboratory, Nairobi and Kisumu	TAC*	*Bordetella pertussis*
		*Chlamydia pneumoniae*
		*Haemophilus influenzae* (all types)
		*Klebsiella pneumoniae*
		*Legionella*
		*Mycobacterium tuberculosis*
		*Mycoplasma pneumoniae*
		Pan-*Salmonella*
		*Streptococcus,* Group A
		*Streptococcus pneumoniae*
		Adenovirus
		Enterovirus
		Human coronavirus 229E/NL63
		Human coronavirus OC43/HKU1
		Human metapneumovirus
		Influenza A
		Influenza B
		MERS-U/MERS-N
		Parainfluenza virus1
		Parainfluenza virus2
		Parainfluenza virus3
		Respiratory syncytial virus
		Rhinovirus
**County**		
Kakuma, Nakuru, and Nyeri	Automated blood culture system	Bacterial pathogens

**Abbreviations:** KEMRI = Kenya Medical Research Institute; MERS = Middle East respiratory syndrome; PCR = polymerase chain reaction; TAC = TaqMan array card.

*Specimen types that can be tested include respiratory specimens (e.g., nasopharyngeal/oropharyngeal swabs, sputum, and bronchoalveolar lavage).

## Improving Response Capacity

**Trainings to support workforce development.** CDC has supported extensive competency-based training for workforce development in Kenya through FELTP,** a field epidemiology training program. Through a collaboration between the Kenya MoH and CDC, the Kenya FELTP^††^ was established within the Kenya MoH in 2004, with a mandate to increase epidemiologic capacity through development of a skilled public health workforce that supports disease surveillance systems and public health emergency responses. After implementation of DaRRE, the Kenya FELTP developed and added a respiratory disease outbreak curriculum, now offered to public health practitioners and FELTP fellows via an annual workshop. Since 2016, the Kenya FELTP infectious diseases elective course has covered principal pathogens that can cause respiratory disease outbreaks and concepts that are important for early detection and response to respiratory events. As of April 2020, four Kenya FELTP participant groups had completed the course (Table 2). In addition, training on respiratory diseases outbreak investigation and public health response was offered to surveillance officers at all SARI sites. Hospital personnel (clinicians and nurses) and public health practitioners from county health departments also attended the course trainings (Table 2).

**TABLE 2:** Summary of trainings to support workforce development — CDC Kenya Detection and Response to Respiratory Events Strategy (DaRRE), April 2016–April 2020

Type of training	Personnel trained	Training site	No. of trainings provided	No. of persons trained
FELTP infectious diseases elective respiratory session on DaRRE*	FELTP fellows	Ministry of health facilities in Nairobi	4	80
Influenza surveillance and DaRRE^†^	SARI surveillance officers	Kakamega, Kakuma, Marsabit, Mombasa, Nakuru, and Nyeri counties; Kenyatta National Hospital	2	75
Influenza surveillance and acute febrile illness	SARI surveillance officers	Kakamega, Kakuma, Marsabit, Mombasa, Nakuru, and Nyeri counties; Kenyatta National Hospital	1	35
Bacteriology for respiratory pathogens	Laboratory technicians	Kakuma, Kitale, Nakuru, Nyeri, and Thika counties; KEMRI laboratories at Kisumu	2	20
Assessor training on the Antimicrobial Resistance Laboratory Quality scorecard	Laboratory technicians	Nakuru, Nyeri, and Thika counties	1	10
Integrated disease surveillance with influenza surveillance	Public health officials, county disease surveillance officers, and clinicians	Kakamega, Marsabit, Mombasa Nakuru, Nyeri, and Thika counties	2	80
Event-based surveillance^†^	National and county trainers of trainers	Nairobi; Nakuru and Siaya counties	2	70
	Community and animal health assistants	Nakuru and Siaya counties	2	26
	Community health volunteers	Nakuru and Siaya counties	2	397

**Abbreviations:** FELTP = Field Epidemiology and Laboratory Training Program; KEMRI = Kenya Medical Research Institute; SARI = severe acute respiratory infection.

* Areas covered during training included respiratory outbreak investigation, specimen collection, and pathogen-specific topics.

^†^ Topics covered during training included use of electronic reporting, signals to identify priority diseases, principles of event-based surveillance (EBS), and differences between EBS and indicator-based surveillance.

**EOC and hotline.** The EOC and a telephone hotline are important for any public health action in Kenya. Therefore, a 2-week EOC rotation for Kenya MoH officers and FELTP fellows was established in 2017 to monitor media, rumor logs, and calls of public health events of concern (e.g., natural disasters, clusters of severe infectious diseases, and reportable diseases including SARI, polio, cholera, and measles). In addition, DaRRE partners established a toll-free, mobile phone–based hotline in the EOC that is monitored 24 hours each day of the week and allows for expedited notification of such events. Event-based surveillance reports received through the hotline or mobile phone–based electronic reporting system were incorporated into the EOC database and dashboard in real-time. The database will also capture information on county and national public health response (e.g., timeliness, information quality, and usefulness) to inform the government of required resources.

## Discussion

Under the World Health Organization’s IHR, countries are required to strengthen their capacity to respond promptly and effectively to public health events of international concern (*9*). Timely detection of acute respiratory events and an effective, rapid public health response rely on successful integration of multiple systems. DaRRE initiatives, which can be implemented together or in modular format, support IHR requirements and allow for flexibility to fit the country’s needs and available resources. Efforts have been made to fully integrate DaRRE into Kenya’s existing event detection and reporting systems to ensure sustainability.

Since the implementation of DaRRE activities in Kenya, the Kenya MoH effectively responded to several respiratory events. One recent investigation illustrates its success. In July 2019, a hospital physician called the EOC hotline to report increased numbers of persons hospitalized with an unknown severe respiratory illness, including two patients who had died. The Kenya MoH initiated a prompt response and FELTP teams were deployed to the field within 24 hours. Respiratory specimens were collected and tested at the National Influenza Center and at the Kenya Medical Research Institute laboratory where TAC was used. The patients who died and others associated with this cluster were found to be infected with influenza A/H1N1pdm09 virus, the pathogenic cause of the 2009 influenza pandemic. Patient cohorting was implemented and hospital personnel were advised regarding recommended infection control practices. Evaluation of the community event-based surveillance pilot is planned to begin in September 2020.

Severe respiratory disease clusters are often poorly investigated, and disease etiology is not well understood, limiting capacity to respond appropriately (*10*). This is especially challenging in resource-limited settings. Targeted investments and timely detection and response to acute respiratory disease clusters are, therefore, key for the GHSA. During the current COVID-19 pandemic, Kenya has been able to leverage resources that were strengthened through DaRRE to detect and respond to COVID-19 cases. DaRRE provides an integrated approach and capitalizes on existing resources that could lead to sustainable improvement in public health preparedness and might be a model for other countries in the region.

**Summary**

**What is already known about this topic?**

Severe respiratory disease clusters are challenging to investigate in resource-limited settings, and disease etiology is not always identified; these factors might limit the capacity to respond to respiratory disease clusters.

**What is added by this report?**

In 2016, Kenya was selected to pilot the Detection and Response to Respiratory Events (DaRRE) strategy, developed by CDC to enhance country capacity for identifying and controlling respiratory disease outbreaks. During April 2016–April 2020, laboratory capacity, surveillance, and emergency operations were strengthened in Kenya.

**What are the implications for public health practice?**

DaRRE can support International Health Regulation requirements and is sufficiently flexible to accommodate country needs and available resources.

## Footnotes

* G7 is a group of seven industrialized nations (Canada, France, Germany, Italy, Japan, the United Kingdom, and the United States) that meets annually to discuss issues such as global economic governance, international security, and energy policy.

† GHSA is an effort by a group of countries, international organizations, nongovernmental organizations, and private sector companies with the goals of accelerating progress toward a world safe and secure from infectious diseases threats, promoting global health security as an international priority, and progressing towards full implementation of global health security frameworks, such as the World Health Organization International Health Regulations https://www.cdc.gov/globalhealth/healthprotection/ghs/pdf/ghsa-action-packages_24-september-2014.pdf.

§ https://www.who.int/ihr/publications/9789241580496/en/.

¶ https://www.cdc.gov/globalhealth/security/ghsa5year/cdc-5-years-ghsa.html.

** https://www.cdc.gov/globalhealth/healthprotection/fetp/index.htm.

†† https://www.tephinet.org/training-programs/kenya-field-epidemiology-and-laboratory-training-program.
